# Hidden Risks of Miniscrew-Assisted Rapid Palatal Expansion (MARPE): A Retrospective Analysis of Treated Cases With Preventive Strategies

**DOI:** 10.7759/cureus.93240

**Published:** 2025-09-25

**Authors:** Rajalakshmi SJ, Nausheer Ahmed, Niharika Eduru, Deeksha YN, Kesari Ravi, Gowri Sankar Singaraju

**Affiliations:** 1 Orthodontics and Dentofacial Orthopedics, Government Dental College and Research Institute Bangalore, Bengaluru, IND; 2 Orthodontics and Dentofacial Orthopedics, Narayana Dental College, Nellore, IND

**Keywords:** appliance, breakage, complications, expansion, fracture, marpe, miniscrew, mucositis, orthodontic, swallowing

## Abstract

Introduction: Miniscrew-assisted rapid palatal expansion (MARPE) is an effective non-surgical alternative for managing transverse maxillary deficiency in late adolescents and young adults. While its skeletal effects are well documented, reports of clinical complications remain limited. This retrospective study presents a comprehensive case-based analysis of MARPE-related complications within a cohort, highlighting both management outcomes and preventive strategies.

Materials and methods: A retrospective review was conducted of 24 consecutive MARPE cases (mean age 17.4 ± 1.3 years; 11 males, 13 females) treated between January and December 2024. Appliance designs included a two-screw hybrid MARPE (n = 16) and a four-screw maxillary skeletal expander (MSE) (n = 8), with miniscrews measuring 1.8-2.0 mm in diameter and 9-11 mm in length. Data were collected on demographics, appliance type, screw size, fabrication method (custom vs. prefabricated), complications, management, and treatment interruptions. In addition to summary statistics, six representative case studies of complications were described in detail.

Results: Of the 24 patients, eight (33.3%) developed true complications, two (8.3%) experienced procedural delays due to screw-locking defects, and 14 (58.3%) had no adverse events. Mechanical complications (n = 6) included screw fracture, appliance arm breakage, and acrylic failure, while biological complications (n = 2) involved tongue irritation and peri-implant mucositis. When the complication and no-complication groups were compared, an independent t-test revealed no significant age difference (17.8 vs. 17.1 years, p = 0.21). Chi-square analysis revealed no significant gender effect, although males experienced more complications (54.5% vs. 30.8%, OR = 2.7, p = 0.064). Fisher’s exact test demonstrated that complications were confined to screws ≤10 mm, while none occurred with 11 mm or 2.0 × 10 mm screws (p = 0.03). Activation was interrupted for at least two weeks until the issue was resolved. Six detailed case reports illustrate the causes, management, and clinical precautions for these events.

Conclusions: MARPE is an effective and minimally invasive alternative to surgically assisted rapid palatal expansion, but complications occur in nearly one-third of cases. These may arise with any appliance type and are often related to human or technical errors. Early recognition, implementation of preventive strategies, and adherence to standardized protocols are crucial for ensuring safe and predictable outcomes.

## Introduction

Transverse maxillary deficiency is a dentofacial anomaly characterized by a constricted maxillary arch relative to the mandible, presenting clinically as posterior crossbite, crowding, or airway obstruction [1,2]. In growing patients, orthopedic expansion is reliable; however, in adults, progressive ossification and interdigitation of the midpalatal suture reduce the effectiveness of conventional rapid palatal expansion (RPE) [3].

Angell first described the splitting of the midpalatal suture in 1860 [4], but the technique was not widely accepted until Haas popularized RPE in the 1960s using tooth-borne acrylic appliances [5]. While RPE became the standard in children, skeletally mature patients exhibited limited skeletal expansion and undesirable dental effects such as buccal tipping and periodontal compromise. Surgically assisted rapid palatal expansion (SARPE) was introduced to overcome these limitations but carries surgical risks, postoperative morbidity, and higher costs [6].

Miniscrew-assisted rapid palatal expansion (MARPE) was developed as a non-surgical alternative for late adolescents and adults. Lee et al. first demonstrated its effectiveness [7], and subsequent refinements by Moon, MacGinnis et al., and Suzuki et al. introduced skeletal-anchored maxillary expanders with bicortically engaged miniscrews to optimize orthopedic forces and reduce dental side effects [8-10]. MARPE has since been validated as a minimally invasive and effective option [11-13], with reported advantages including greater skeletal expansion, reduced periodontal compromise, and avoidance of surgical risks [6,10,12].

Nevertheless, limitations and risks exist. Reported complications include incomplete expansion in adults with advanced sutural ossification [3], residual dentoalveolar side effects [6], peri-implant mucositis, screw loosening, and, rarely, treatment failure requiring SARPE [14-18]. Despite the increasing global use of MARPE, systematic analyses of complications remain scarce, and most reports focus on skeletal and dental outcomes rather than iatrogenic events.

This retrospective clinical study was designed to address that gap. We analyzed 24 consecutive MARPE patients treated in a single institution, documenting the prevalence and types of complications. Six representative cases are presented in detail to illustrate clinical scenarios, management, and outcomes. Furthermore, preventive strategies are outlined to help clinicians minimize risks and enhance the predictability and safety of MARPE therapy.

## Materials and methods

This study was a retrospective review of patients treated in the Department of Orthodontics at the Government Dental College and Research Institute, Bengaluru, India. All patients (or their guardians) provided informed consent for use of their anonymized clinical records and images for scientific and educational purposes. As this was a retrospective analysis of de-identified data with no additional intervention, formal institutional ethical approval was not required.

All consecutive patients who underwent MARPE between January and December 2024 were included in the study. The inclusion criteria were patients aged 15 years or older who were treated with either hybrid or bone-borne miniscrew-supported expanders and had achieved full expansion as per treatment objectives. No a priori sample size calculation was performed, as the study encompassed all eligible cases within the one year.

For each patient, the records included age, sex, appliance type, fabrication method, miniscrew dimensions, occurrence and type of complication, management rendered, and whether treatment was interrupted. Complications were categorized broadly as mechanical, biological, or procedural delays. Mechanical complications included events such as screw fracture, appliance arm breakage, or acrylic failure. Biological complications referred to patient-reported symptoms such as mucositis, tongue irritation, or pain that interfered with function. Procedural delays, such as screw-locking issues, were not considered true complications but were noted when they occurred. Any event that required suspension of appliance activation for more than two weeks was considered a major complication.

All complications were managed according to appropriate clinical protocols, and the details of management were documented in the patient records.

Statistical analysis

Data were entered into Microsoft Excel (Microsoft Corp., Redmond, WA, USA) and analyzed using SPSS Statistics version 25.0 (IBM Corp., Released 2017. IBM SPSS Statistics for Windows, Version 25.0. Armonk, NY: IBM Corp.). All variables, including age distribution, gender, appliance type, fabrication method, screw size, and treatment interruptions, were treated as categorical data and expressed as frequencies and percentages. Associations between these variables and the occurrence of complications were assessed using Fisher’s exact test, as several cell counts were small. Odds ratios (OR) with 95% confidence intervals (CI) were calculated for key comparisons (e.g., gender and screw size). A p-value < 0.05 was considered statistically significant.

## Results

A total of 24 patients were included, with a mean age of 17.4 years (SD 1.3; range, 15.4-19.6). The cohort comprised 11 males (45.8%) and 13 females (54.2%). Sixteen patients (66.7%) were treated with a hybrid two-screw MARPE, and eight (33.3%) with a four-screw maxillary skeletal expander (MSE). The majority of appliances were custom-fabricated (83.3%). The miniscrews most commonly used were 1.8 × 10 mm (45.8%), followed by 1.8 × 11 mm (25.0%), 1.8 × 9 mm (16.7%), and 2.0 × 10 mm (12.5%) (Tables 1-2).

**Table 1:** Demographic and cohort characteristics of the patients treated with MARPE appliances * continuous variable NA: not applicable, MARPE: miniscrew-assisted rapid palatal expansion, MSE: maxillary skeletal expander, SD: standard deviation

Variable	Category	n	%
Total patients	–	24	100
Age (years)*	Mean ± SD	17.4 ± 1.3	NA
Age (years)*	Range	15.4–19.6	NA
Gender	Male	11	45.8
	Female	13	54.2
Appliance type	2-screw MARPE	16	66.7
	4-screw MSE	8	33.3
Screw size used	1.8 × 9 mm	4	16.7
	1.8 × 10 mm	11	45.8
	1.8 × 11 mm	6	25.0
	2.0 × 10 mm	3	12.5
Custom vs. prefabricated appliances	Custom	20	83.3
	Prefabricated	4	16.7
Complication types	Mechanical	7	29.1
	Biological	3	12.5
Overall complications		10	41.6
Treatment interruption ≥2 weeks	True complications	6	25.0
	Procedural delays	2	8.3
Total interruptions ≥2 weeks		8	33.3

**Table 2:** Descriptive data of the MARPE patients (anchor type, screw size, and complications) MARPE: miniscrew-assisted rapid palatal expansion, MSE: maxillary skeletal expander

Anchor type	Screw size (diameter × length)	No. of patients (n)	Custom vs. prefabricated (n)	Complications (n)	Complication type(s)
2-screw (hybrid MARPE)	1.8 × 9 mm	3	Custom = 2, prefab = 1	3	Mechanical (screw failures, expansion arm breakage)
	1.8 × 10 mm	8	Custom = 7, prefab = 1	4	Mechanical (screw failures, acrylic issue)
	1.8 × 11 mm	4	Custom = 3, prefab = 1	0	–
	2.0 × 10 mm	1	Custom = 1	0	–
	Total	16	Custom = 13, prefab = 3	7	Mostly mechanical
4-screw (MSE-type)	1.8 × 9 mm	1	Prefab = 1	1	Biological (tongue irritation)
	1.8 × 10 mm	3	Custom = 3	2	Mechanical and biological (pain and discomfort) (mixed)
	1.8 × 11 mm	2	Custom = 2	0	–
	2.0 × 10 mm	2	Custom = 2	0	–
	Total	8	Custom = 7, prefab = 1	3	Mix of mechanical and biological (pain and discomfort)
Overall total		24	Custom = 20, prefab = 4	10 (41.6%)	7 mechanical, 3 biological

Complications were observed in 10 patients (41.6%). Mechanical failures such as screw fracture, appliance arm breakage, or acrylic loss were the most frequent, occurring in seven patients (29.1%). Biological complications, including tongue irritation and peri-implant mucositis, occurred in three patients (12.5%). In addition, two patients (8.3%) experienced procedural delays related to driver or screw-locking defects; these were not classified as true complications. Treatment was interrupted for more than two weeks in eight patients (33.3%), of which six were due to true complications and two were due to procedural delays.

Complications were more prevalent in the two-screw hybrid MARPE group, where seven of sixteen patients were affected, compared with three of eight in the four-screw MSE group. Events were also more frequent with smaller screw sizes, particularly 1.8 × 9 mm and 1.8 × 10 mm, whereas no failures were recorded with 1.8 × 11 mm screws.

A comparative statistical analysis was conducted to evaluate the associations between patient and appliance variables and the occurrence of complications. The results are summarized in Table 3. Males had higher odds of complications than females (OR = 2.7; 95% CI: 0.47-15.4), though the difference was not statistically significant. Appliance type and fabrication method showed no significant effect. However, screw length ≤10 mm was significantly associated with higher complication rates (p = 0.03).

**Table 3:** Comparison of demographic and appliance-related variables between patients with and without complications * statistically significant at p < 0.05. OR with 95% CI calculated using Fisher’s exact test. For screw size, no complications occurred in screws ≥11 mm; therefore, ORs could not be reliably estimated. CI: confidence interval, OR: odds ratio, MARPE: miniscrew-assisted rapid palatal expansion, MSE: maxillary skeletal expander

Variable	Categories	Complications (n = 10)	No complications (n = 14)	OR (95% CI)	Test	p-value
Age (years, mean ± SD)	NA	17.8 ± 1.2	17.1 ± 1.4	NA	t-test	0.21
Gender	Male (n = 11)	6 (54.5%)	5 (45.5%)	2.7 (0.47-15.4)	Chi-square	0.64
	Female (n = 13)	4 (30.8%)	9 (69.2%)			
Appliance type	2-screw MARPE (n = 16)	7 (43.8%)	9 (56.2%)	1.3 (0.2-7.6)	Fisher’s	0.69
	4-screw MSE (n = 8)	3 (37.5%)	5 (62.5%)			
Fabrication	Custom (n = 20)	8 (40.0%)	12 (60.0%)	0.7 (0.09-5.7)	Fisher’s	1.00
	Prefabricated (n = 4)	2 (50.0%)	2 (50.0%)			
Screw size	≤10 mm (n = 15)	8 (53.3%)	7 (46.7%)	Reference	Fisher’s	0.03*
	11 mm (n = 6)	0 (0.0%)	6 (100.0%)	Not estimable		
	2.0 × 10 mm (n = 3)	0 (0.0%)	3 (100.0%)	Not estimable		

The following six cases illustrate the spectrum of complications observed in our cohort, their clinical causes, and management approaches (Table 4) [19-27].

**Table 4:** Complications associated with MARPE: causes, management, and preventive strategies GI: gastrointestinal, CBCT: cone beam computed tomography, MARPE: miniscrew-assisted rapid palatal expansion

Case no.	Complication	Causes	Management	Preventive/precautionary strategies
1	Accidental swallowing of the connecting rod	Detachment of the movable rod during removal; topical anesthesia reduced the gag reflex	Immediate intraoral check; radiograph confirmed swallowed rod; observation only; rod passed naturally in 48 hours, and resolution reconfirmed at seven days	Secure small parts during removal (e.g., floss, gauze); avoid topical anesthesia near the oropharynx; use throat screens; educate patients to report any foreign body sensation [19–21]
2	Post-expansion appliance arm breakage	Stress fatigue at the welded joint; limited support in the two-screw design	Broken arm removed; custom retention plate delivered; stable outcome in retention	Monitor appliance integrity; prefer four-screw systems; reinforce retention with plates/archwires [7,9]
3	Appliance irritation/tongue swelling	Sharp anterior screw extensions contacting the tongue; tongue-thrust habit	Chairside smoothing with finishing bur; protective wax placed; warm saline rinses and dietary advice; resolved in one week	Inspect appliances for sharp edges; identify tongue habits; encourage prompt reporting of discomfort [22,23]
4	Accidental swallowing of a miniscrew	Non-locking/manual driver without secure retention; poor driver–screw engagement; semi-reclined posture	Radiograph confirmed screw in GI tract; observation only; screw passed naturally in 48 hours; clearance reconfirmed at seven days	Use locking/magnetic drivers; ensure secure screw engagement; keep patient upright; employ gauze throat screens [24]
5	Fractured miniscrew during insertion	High palatal bone density; excess torque/poor technique; undersized screw or structural flaw	Procedure halted; minimally invasive retrieval with micro-forceps; antibiotics and analgesics prescribed; further screw placement deferred	Preoperative CBCT to assess bone density; pilot drilling in dense bone; use torque-limiting drivers; select appropriate screw size [24,27]
6	Peri-implant mucositis	Poor hygiene around miniscrews; plaque retention in the anterior palate	Cleaning with Betadine swabs, chlorhexidine rinses, and hygiene reinforcement resulted in inflammation resolution; the appliance remained stable at the six-month follow-up	Emphasize strict hygiene protocols, regular checks during activation, early antiseptic use at the first signs, and maintaining the mechanical stability of the appliance [25,26]

Case 1: accidental swallowing

**Figure 1:**
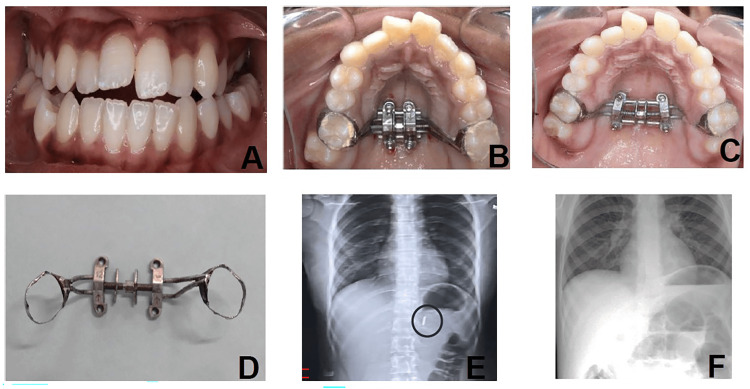
Case 1: Accidental swallowing of the MSE connecting rod. (A) Transverse maxillary deficiency with bilateral crossbite. (B) Insertion of MSE with four screws. (C) Midline diastema with MSE. (D) Autodetached MSE connecting rod. (E) Abdominal radiograph immediately after swallowing showing a swallowed connecting rod. (F) Seven-day post-ingestion abdominal radiograph confirming the absence of the connecting rod. MSE: maxillary skeletal expander

A 19-year-old female with transverse maxillary deficiency underwent skeletal expansion with a four-screw prefabricated MSE. During appliance removal, the anterior connecting rod detached intraorally and was accidentally swallowed. The incident occurred shortly after a topical anesthetic spray was applied to the posterior palate to alleviate discomfort associated with screw removal. This diminished the gag reflex, causing the patient to remain unaware of the swallowed component and preventing protective coughing or choking (Figure 1).

An immediate intraoral examination confirmed the detachment of the rod, and the patient was advised to undergo abdominal radiography. The radiograph confirmed the presence of the swallowed rod, and the patient was closely monitored. She remained asymptomatic, and the rod passed spontaneously through the stools within 48 hours. A follow-up radiography at seven days reconfirmed complete clearance.

Case 2: post-expansion appliance arm breakage

**Figure 2:**
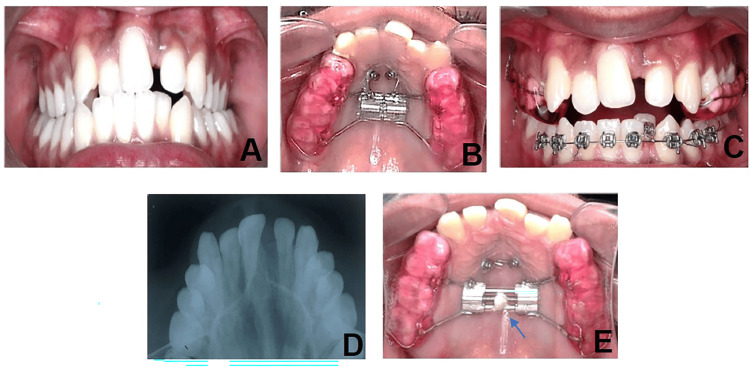
Case 2: Post-expansion appliance arm breakage. (A) Clinical presentation showing transverse maxillary deficiency, (B) MARPE appliance with two miniscrews, (C) clinical confirmation of expansion, (D) radiographic confirmation of expansion, (E) post-expansion fracture of the appliance arm. MARPE: miniscrew-assisted rapid palatal expansion

An 18-year-old male with skeletal Class I malocclusion and transverse maxillary deficiency was treated with a two-screw custom-made MARPE appliance placed in the paramedian anterior palate. Following successful confirmation of the desired expansion, the patient reported that the appliance was mobile during follow-up. Clinical and radiographic examination revealed a fracture of the metallic extension arm connecting the expander to a mini-implant (Figure 2).

The fracture was attributed to stress fatigue at the welded joint, exacerbated by functional forces such as the action of the cheek musculature and the elastic recoil of expanded tissues. The risk is higher in two-screw designs due to limited support. Since expansion was already complete, the broken component was removed, and a custom retention plate was delivered to preserve the achieved correction. The patient remained stable during retention.

Case 3: appliance irritation/tongue swelling

**Figure 3:**
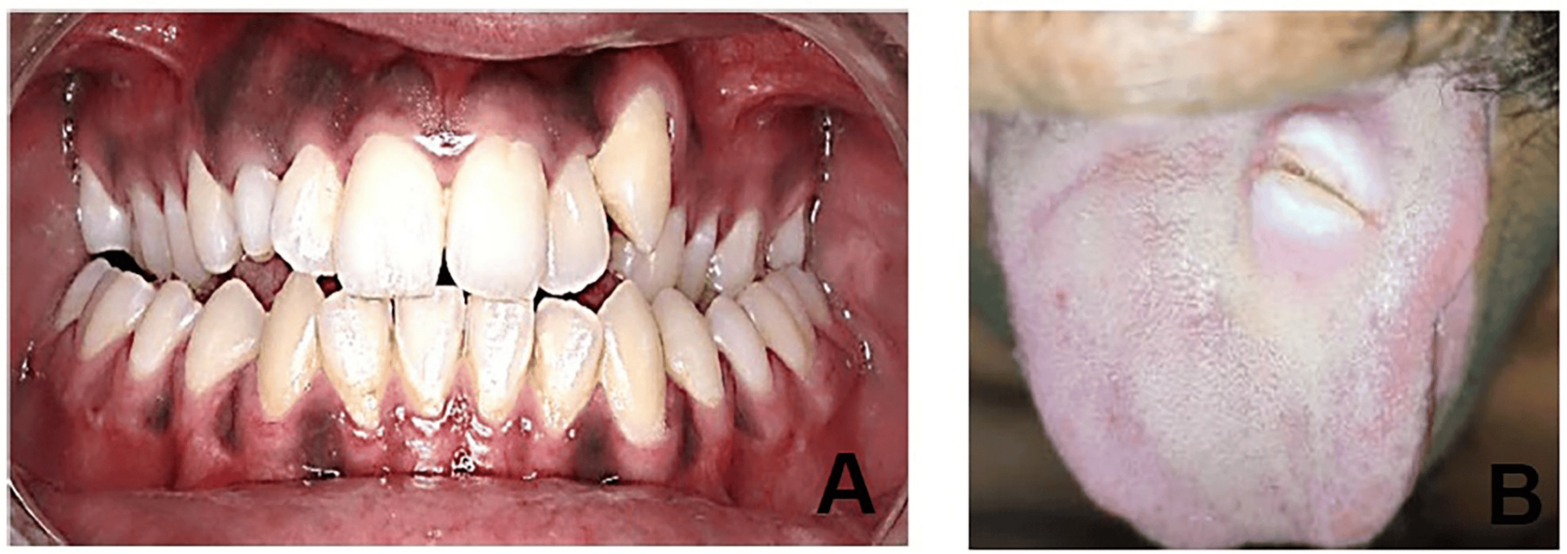
Case 3: Appliance irritation with tongue swelling. (A) Transverse maxillary deficiency with bilateral posterior crossbite. (B) swelling on the dorsum of the tongue in contact with MARPE screw extensions. MARPE: miniscrew-assisted rapid palatal expansion

A 17-year-old male undergoing expansion with MARPE presented with soreness of the tongue during the second week of activation. Examination revealed a localized swelling on the dorsum of the tongue, directly contacting the anterior screw extensions of the appliance. The swelling was mildly tender but showed no signs of ulceration or infection (Figure 3).

The irritation was attributed to direct contact of the tongue with sharp or exposed screw edges, which was aggravated by an existing mild tongue-thrusting habit, resulting in increased pressure and frequency of contact during speech and swallowing. A chairside adjustment was performed to smooth sharp edges, and orthodontic wax was applied to protect the soft tissues. The patient was advised to avoid spicy foods, use warm saline rinses, and maintain hygiene. The swelling resolved within one week without further complications.

Case 4: screw dislodgement during placement and accidental swallowing

**Figure 4:**
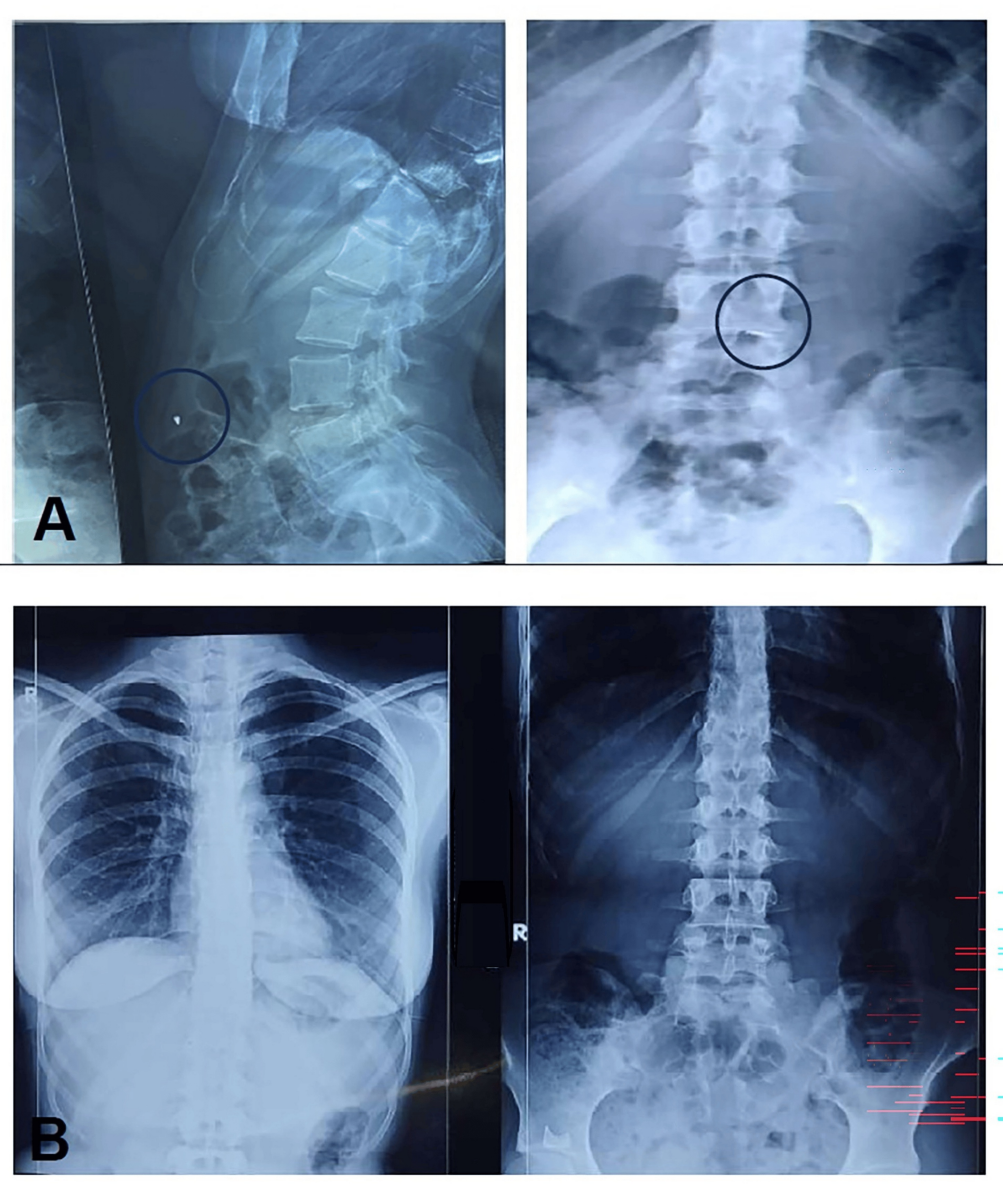
Case 4: Accidental swallowing of a miniscrew. (A) Immediate abdominal radiograph showing miniscrew in the lower abdomen. (B) Seven-day post-ingestion abdominal radiograph confirming absence of the miniscrew.

During placement of a custom-made two-MARPE appliance in a 17-year-old female with skeletal Class I malocclusion, a palatal miniscrew (2 × 10 mm) became dislodged from the driver before engaging bone and was accidentally swallowed. The event occurred early in the procedure, before the screw could be securely engaged with the bone. The event occurred due to the use of a manual driver without a locking or magnetic mechanism, resulting in poor driver-screw engagement and a momentary loss of control during transfer. The semi-reclined patient posture increased the likelihood of the screw slipping posteriorly (Figure 4).

The patient remained asymptomatic. Radiographs confirmed the screw in the gastrointestinal tract, and observation was chosen as management. The screw passed naturally within 48 hours, and a follow-up radiograph at seven days confirmed clearance. No surgical intervention was required.

Case 5: fractured miniscrew during insertion

**Figure 5:**
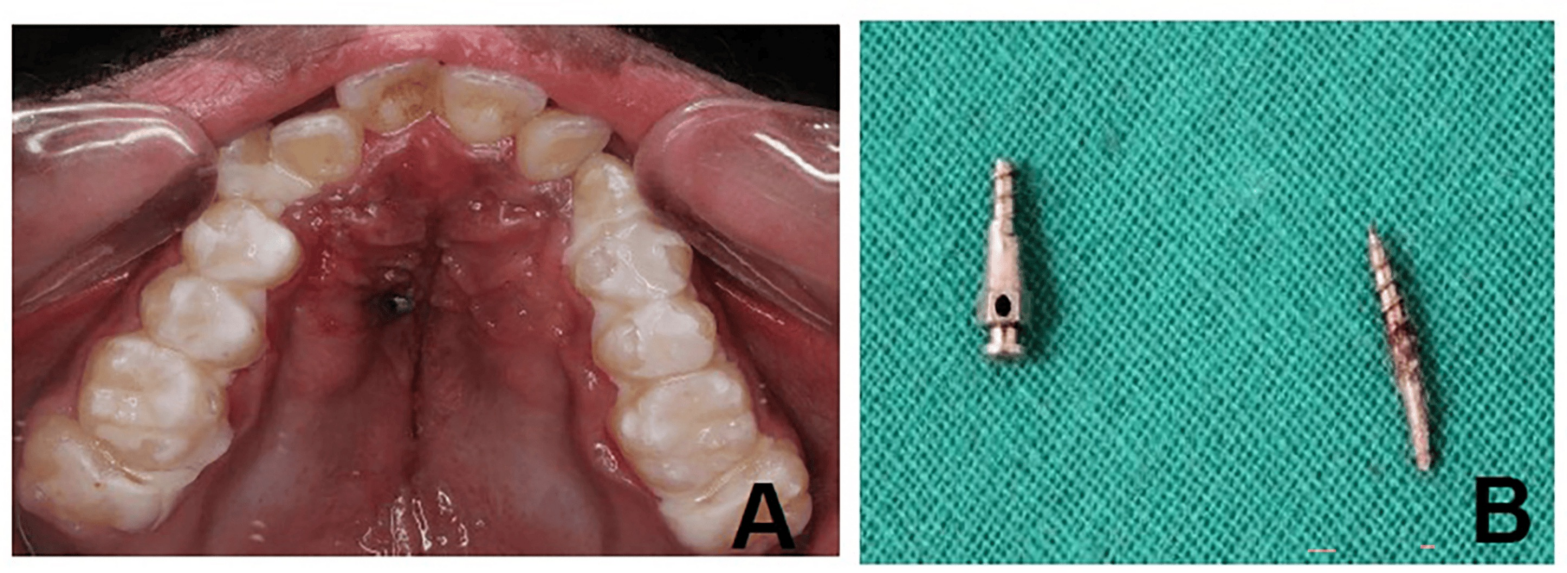
Case 5: Fractured miniscrew during insertion. (A) Intraoral picture showing a fractured miniscrew embedded in the palate. (B) Retrieved a fractured miniscrew after minimally invasive removal.

A 16-year-old male with transverse maxillary deficiency was planned for expansion with a MARPE appliance. During insertion of the anterior right miniscrew (1.8 × 9 mm), resistance was encountered, and despite controlled torque, the screw fractured. The apical half remained embedded in the palatal bone while the coronal fragment detached (Figure 5).

The fracture was attributed to high bone density in the midpalatal region, excessive torque, use of an undersized screw, or a possible structural defect. Conservative retrieval was performed under local anesthesia without flap elevation, using micro-forceps. The patient was prescribed antibiotics and analgesics, and healing was uneventful. Reinsertion was delayed (1.8 x 10 mm) until full recovery was achieved.

Case 6: peri-implant mucositis

**Figure 6:**
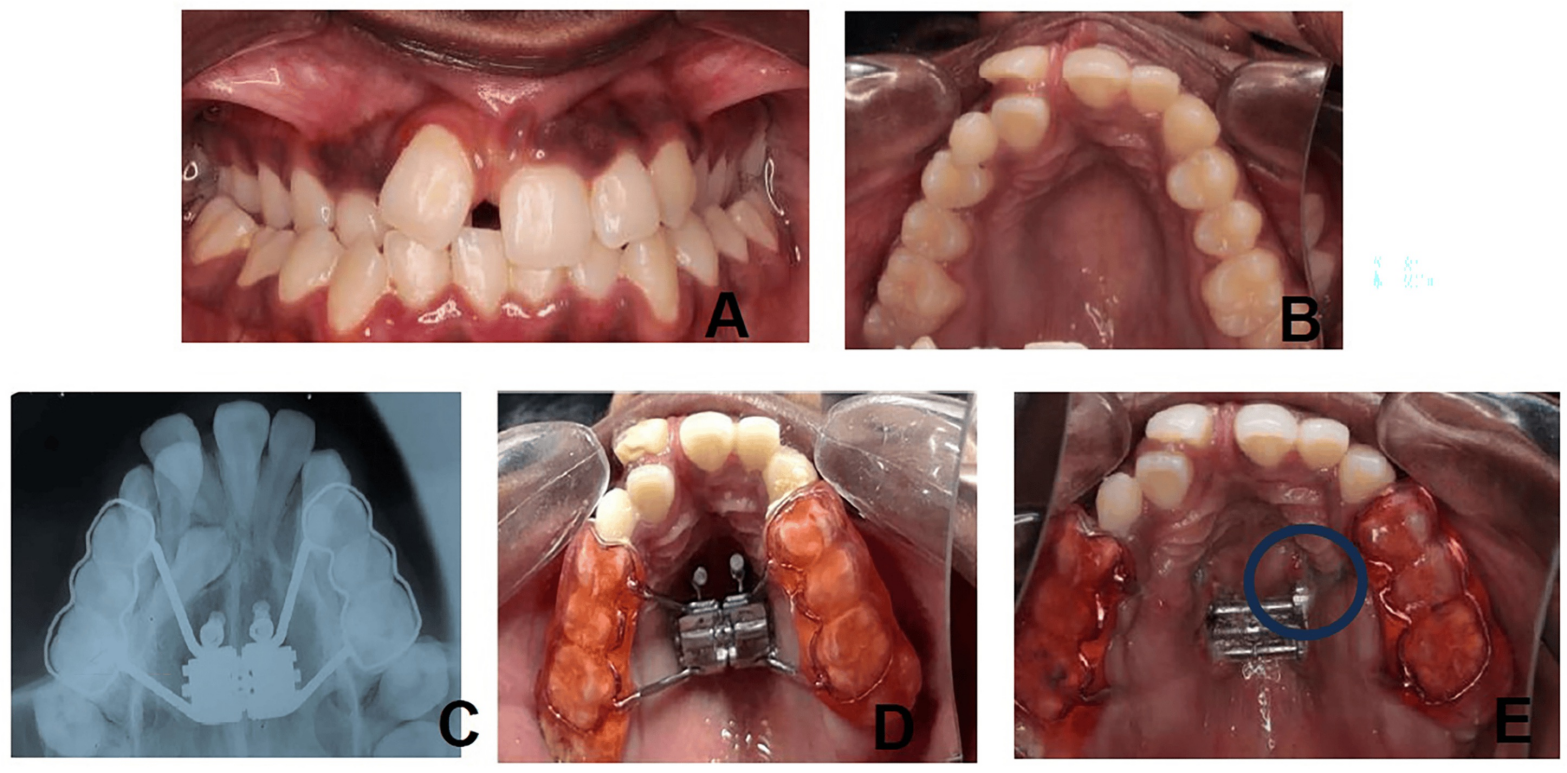
Case 6: Peri-implant mucositis. (A) Bilateral posterior crossbite with constricted maxilla. (B) Radiograph showing MARPE appliance. (C) MARPE appliance with two miniscrews in situ. (D) Soft tissue enlargement around the miniscrew indicating peri-implant mucositis. MARPE: miniscrew-assisted rapid palatal expansion

A 16-year-old female with skeletal Class III malocclusion and transverse maxillary deficiency with posterior crossbite was treated with MARPE supported by two anterior miniscrews. By the second week of activation, she developed swelling and discomfort around the miniscrews, especially on the left side. Examination revealed peri-implant mucositis, though the appliance remained stable with no screw mobility (Figure 6).

The complication was attributed to plaque accumulation and inadequate hygiene in the anterior palate. Management included local cleaning with betadine swabs, chlorhexidine mouth rinses, and reinforcement of oral hygiene practices. The inflammation resolved without interrupting treatment, and the appliance remained stable throughout a six-month follow-up period.

## Discussion

MARPE has emerged as a predictable and minimally invasive alternative to SARPE in late adolescents and young adults [7-10]. Its advantages include greater skeletal expansion with fewer dental side effects than conventional RPE, reduced periodontal damage, and the avoidance of surgical morbidity [11-13]. Nevertheless, as highlighted by recent reviews, complications such as screw failure, appliance breakage, and mucosal irritation are not uncommon and may compromise treatment if not promptly addressed [14,18,19].

In the present series of 24 patients, complications were documented in 41.6% of cases, occurring with both prefabricated and custom-made appliances. Mechanical failures were most frequent, followed by biological complications and rare iatrogenic events such as accidental ingestion. Treatment was interrupted in one-third of patients, although all complications were resolved without long-term sequelae. When baseline characteristics were statistically compared between the complication and non-complication groups, no significant difference was observed in mean age (17.8 vs. 17.1 years, p = 0.21) or gender distribution. However, males showed a higher proportion of complications (54.5% vs. 30.8%, OR ≈ 2.7, p = 0.64). Appliance type (two-screw MARPE vs. four-screw MSE, p = 0.69) and fabrication method (custom vs. prefabricated, p = 1.00) were not significantly associated with complication rates. In contrast, screw size demonstrated a significant effect: complications were confined to screws ≤10 mm, while none occurred with screws ≥11 mm (p = 0.03). Treatment interruptions of ≥2 weeks were observed in 25% of the cohort, caused by both true complications and procedural delays. Notably, delays occurred equally among males and females (27.3% vs. 23.1%), indicating no gender predisposition. This reinforces that preventive strategies such as careful screw selection, activation checks, and monitoring are universally important, regardless of sex. These findings are comparable to earlier reports, which noted that while MARPE is highly effective, adverse events can occur at all stages of treatment, from screw insertion to post-expansion retention [12,13,20].

These observations indicate that MARPE complications are multifactorial, spanning mechanical, biological, and iatrogenic domains. While most events were manageable without long-term effects, they frequently interrupted treatment and highlighted the importance of preventive strategies. To contextualize these findings, the following sections discuss the major categories of complications encountered in this series, relating them to mechanisms described in previous literature and outlining key preventive measures.

Iatrogenic events

In two of our patients, iatrogenic complications were related to loss of appliance components, including accidental ingestion of a MARPE part and intraoral screw displacement during transfer. Both events are consistent with earlier reports [19-21,24], which identified inadequate control of detachable components and suppression of protective reflexes under topical anesthesia as risk factors. Preventive measures include securing small parts with floss or gauze, using locking or magnetic drivers, maintaining the patient in a more upright position, and employing throat screens to minimize the risk of aspiration or ingestion [19-21, 24]. Importantly, clinicians must be prepared for emergency airway clearance protocols, including the Heimlich maneuver in cases of suspected obstruction, followed by immediate intraoral inspection, radiographic confirmation, and a referral for medical management where indicated [21,24].

Other iatrogenic events in our cohort were related to appliance design and surface finish, such as mucosal trauma from unpolished edges in contact with soft tissues. This complication, encountered in one patient, parallels earlier observations that rough or improperly finished appliance surfaces can predispose to tissue injury [22,23]. Additional contributing factors include tongue habits that aggravate localized trauma [23]. Preventive strategies, therefore, involve meticulous inspection and finishing of appliance surfaces before delivery, as well as early recognition and prompt adjustment if irritation occurs. Patient counseling on parafunctional habits and early reporting of discomfort can further reduce the risk of progression.

Mechanical and biological factors

Some complications observed in this series reflected the combined influence of mechanical and biological determinants. Screw displacement during transfer, for example, often arises from inadequate engagement of the driver, unstable patient positioning, or airway vulnerability. Preventive measures include the use of locking or magnetic drivers, secure engagement before transfer, maintaining the patient in a more upright position, and employing throat screens or gauze barriers to reduce the risk of aspiration or ingestion [24]. Tissue-related variables such as gag reflex sensitivity and airway anatomy may further influence the clinical severity of such events.

Similarly, screw fracture is closely linked to mechanical overload but is also modulated by biological variability in palatal bone density and mucosal thickness. Dense cortical bone increases resistance during insertion, while thick mucosa may alter effective bone purchase. Preventive strategies include preoperative CBCT assessment to identify zones of high resistance, pilot drilling when indicated, the use of torque-limiting drivers, and selecting screws of appropriate length and diameter to match the underlying bone and soft tissue environment [24,27]. Taken together, these findings emphasize that careful consideration of both mechanical technique and patient-specific tissue factors is critical to minimizing MARPE-related complications.

Biological complications related to oral hygiene

Inadequate oral hygiene around miniscrews may lead to peri-implant mucositis or early peri-implantitis, compromising stability and patient comfort [25,26]. Effective prevention requires clear patient education, reinforcement during follow-up, and early intervention if inflammation develops. Practical strategies include the use of soft toothbrushes, interproximal aids, and adjunctive antiseptic rinses such as chlorhexidine. Emphasizing hygiene as an integral component of MARPE therapy helps ensure uninterrupted treatment and minimizes biological complications.

Taken together, these six cases demonstrate the diverse spectrum of MARPE-related complications, spanning mechanical, biological, and iatrogenic categories. Mechanical failures predominated, but biological complications and rare events such as accidental ingestion were also encountered. Complications occurred with both prefabricated and custom-made appliances and were more frequent in two-screw hybrid designs than in four-screw MSE appliances [7,9]. Smaller screws (1.8 × 9 mm and 1.8 × 10 mm) were associated with higher complication rates, whereas no failures occurred with 1.8 × 11 mm screws, suggesting that both appliance design and screw dimension influence clinical stability.

The implications of this series extend beyond appliance type, as events were also linked to human and technical factors, including improper handling of detachable components, inadequate torque control during screw insertion, the use of non-locking drivers, and insufficient emphasis on patient hygiene. These operator- and patient-related contributors highlight the importance of preventive strategies in everyday clinical practice.

To date, no direct comparative data are available regarding complication profiles between custom-made and prefabricated MARPE appliances. In our series, complications occurred in both groups, underscoring the need for prospective comparisons to clarify whether appliance type or fabrication method significantly affects the incidence of complications.

The strength of this study lies in its detailed documentation of real-world MARPE complications, supported by case-based analyses, clinical photographs, and radiographs. However, the retrospective design, modest sample size, and descriptive nature of the study limit the generalizability of these observations. The absence of a control group and lack of long-term follow-up further limit conclusions regarding prognosis or stability after complication management. While prospective controlled trials may not be feasible for this purpose, long-term, multicenter studies and collaborative registries could contribute valuable insights into unforeseen complications and their management, thereby strengthening the evidence base for clinical decision-making.

From a clinical perspective, the findings emphasize the need for preventive measures, including secure handling of detachable components, the use of locking or magnetic drivers, reinforcement of appliance arms in two-screw designs, careful screw selection based on bone density, and strict oral hygiene protocols. Early identification and conservative management of complications enabled all patients in this series to complete treatment without long-term sequelae. Future multicenter, prospective studies should aim to quantify the incidence of complications across various age groups, screw sizes, and appliance designs, and to assess long-term stability following the management of complications.

## Conclusions

MARPE represents a minimally invasive and effective alternative to SARPE for correcting transverse maxillary deficiency in young adults. Nevertheless, complications, including accidental ingestion of appliance components, mechanical failures, and soft tissue irritation, were observed in nearly one-third of cases. Early recognition and conservative management are critical to avoid escalation and ensure successful outcomes. Importantly, complications may arise with any MARPE appliance, whether custom or prefabricated, hybrid or bone-borne, and are often related to human or technical errors such as improper handling, inadequate torque control, or insufficient hygiene reinforcement. Preventive strategies, including careful case selection, preoperative imaging, preference for four-screw expanders, secure handling of components with locking drivers or barriers, and strict oral hygiene protocols, substantially reduce the risk. With appropriate operator training, patient education, and adherence to standardized protocols, MARPE can deliver predictable and safe results, reinforcing its role as a reliable tool in contemporary orthodontic practice.
